# Design of the SALT Osteoporosis Study: a randomised pragmatic trial, to study a primary care screening and treatment program for the prevention of fractures in women aged 65 years or older

**DOI:** 10.1186/s12891-017-1783-y

**Published:** 2017-10-27

**Authors:** P. J. M. Elders, T. Merlijn, K. M. A. Swart, W. van Hout, B. C. van der Zwaard, C. Niemeijer, M. W. Heymans, A. A. van der Heijden, F. Rutters, H. E. van der Horst, P. Lips, J. C. Netelenbos, N. M. van Schoor

**Affiliations:** 1 Department of General Practice and Elderly Care Medicine, VU University Medical Center, Amsterdam Public Health research institute, Amsterdam, The Netherlands; 2 Stichting ArtsenLaboratorium en Trombosedienst, Koog aan de Zaan, The Netherlands; 3 Department of Epidemiology and Biostatistics, VU University Medical Center, Amsterdam Public Health research institute, Amsterdam, The Netherlands; 4 Department of Internal Medicine, Endocrine Section, VU University Medical Center, Amsterdam Public Health research institute, Amsterdam, The Netherlands

**Keywords:** Osteoporosis, Fractures, Primary care, Randomised pragmatic trial, Screening, Bisphosphonates

## Abstract

**Background:**

Several drugs have become available for the treatment of osteoporosis. However, screening and treatment of patients with a high fracture risk is currently not recommended in the Netherlands, because the effectiveness of bone sparing drugs has not been demonstrated in the general primary care population. Here we describe the design of the SALT Osteoporosis study, which aims to examine whether the screening and treatment of older, female patients in primary care can reduce fractures, in comparison to usual care.

**Methods:**

A randomised pragmatic trial has been designed using a stepwise approach in general care practices in the Netherlands. Women aged ≥65 years, who are not prescribed bone sparing drugs or corticosteroids are eligible for the study. First, women with at least one clinical risk factor for fractures, as determined by questionnaires, are randomly assigned to the intervention or control group. Second, women in the intervention group having a high fracture risk according to our screening program, including an adapted fracture risk assessment (FRAX) tool, combined with dual-energy x-ray absorptiometry (DXA), and instant vertebral assessment (IVA), are offered a structured treatment program. The women in the control group receive care as usual and will undergo the same screening as the intervention group at the end of the trial. The follow-up duration will be three years and the primary outcome is time to first incident fracture and the total number of fractures.

**Discussion:**

The results of the current study will be very important for underpinnings of the prevention strategy of the osteoporosis guidelines.

**Trial registration:**

ID NTR2430. Registered 26 July 2010.

## Background

Osteoporosis is a chronic and multifactorial disorder, which is characterized by low bone mass and micro-architectural deterioration of bone tissue, resulting in fractures after minimal or moderate trauma [1]. Fractures of the distal forearm, the vertebrae and the hip are the most serious consequences of osteoporosis [2, 3]. In the Netherlands, more than 27,000 distal forearm fractures, 6700 vertebral fractures, 17,000 hip fractures, and about 67,000 other fractures occur among persons aged >50 years every year, of which 32% can be attributed to osteoporosis [3]. Osteoporotic fractures are associated with increased morbidity and mortality for many years after the fracture, decreased quality of life and high costs for the society [3, 4].

Several drugs have become available, which have been shown to reduce the risk of fractures with 20-50% in controlled settings [5–8]. Optimal strategies to identify persons at high risk of osteoporotic fractures, and to prevent such fractures have been under discussion for a long time. The guidelines for treating osteoporosis vary strongly between different countries, and range from a conservative approach to almost complete screening of post-menopausal women aged >65 years with dual-energy x-ray absorptiometry (DXA) or even treatment of patients with fragility fractures without additional bone densitometry measurements [9–15]. The Dutch guidelines for general practitioners (GP’s) advocate a conservative approach and advise a stepwise approach which identifies patients with a high fracture risk, by using both clinical risk factors for fractures, which in case of a high risk score is followed by a DXA measurement, and the assessment of prevalent vertebral fractures (see Table 1) [11]. Subsequently, patients at risk with a T-score < −2.5 or with a prevalent vertebral fracture should be treated with bone sparing medication, such as bisphosphonates. The most important reason for this conservative approach is that only few patients will be treated unnecessarily. However, as a consequence, a large part of patients with an increased fracture risk will not be treated. A second reason for this conservative approach is the fact that the effectiveness of the treatment with bisphosphonates has mainly been shown in individuals with known low bone mineral density [5–8], and not in a general primary care population.

**Table 1:** Risk factors for fractures according to the Dutch guideline for general practitioners (GP) for primary osteoporosis

Risk factor	Risk score
Vertebral fracture	4
Recent fracture (<2 years) after the age of 50 years	4^a^
Age ≥ 70 years	1
Age ≥ 60 years	1
Non recent fracture after the age of 50 years	1
Additional non recent fracture after the age of 50 years at a separate occasion	1
Parental hip fracture	1
Body weight < 60 kg	1
Severe immobility or 1 fall or more in the last year	1

Bone densitometry and morphological assessment of vertebral fractures (unless a vertebral x-ray has already been performed) is indicated if the total risk score is ≥4 points. Subsequently bisphosphonate treatment for 5 years is advised if the bone mineral density (BMD) of either femoral neck or lumbar spine shows a T-score ≤ −2.5 or if a prevalent vertebral fracture (≥25% height reduction) is present^a^

^a^In October 2012, when the trial had already started, the Dutch guidelines for GP’s for primary osteoporosis were updated [11]. In the previous guidelines, the treatment threshold of BMD (femoral neck or spine) was a T- score < −2.5 for patients aged <70 years, or a Z-score ≤ −1 for patients aged ≥70 years

In the last decade, the need for more pragmatic designs of clinical trials has been recognized, and pragmatic designs have been emerging [16]. Pragmatic trials are designed to study the effectiveness of an intervention in routine clinical practice. They are characterized by a high heterogeneity, with respect to patients, treatments, and clinical settings, which results in a high external validity of the findings [16]. Because the effects of treatment with bisphosphonates have been demonstrated in controlled settings only, there is a need for more pragmatic studies in primary care in which patients with a high fracture risk are identified and the effect of the bone sparing treatment is examined. The objective of the current study is to describe the design of the SALT Osteoporosis Study aiming to examine the effect of screening and treatment of patients with a high fracture risk in primary care on fracture incidence in a pragmatic setting.

## Methods/design

### Trial design and participants

**Fig. 1:**
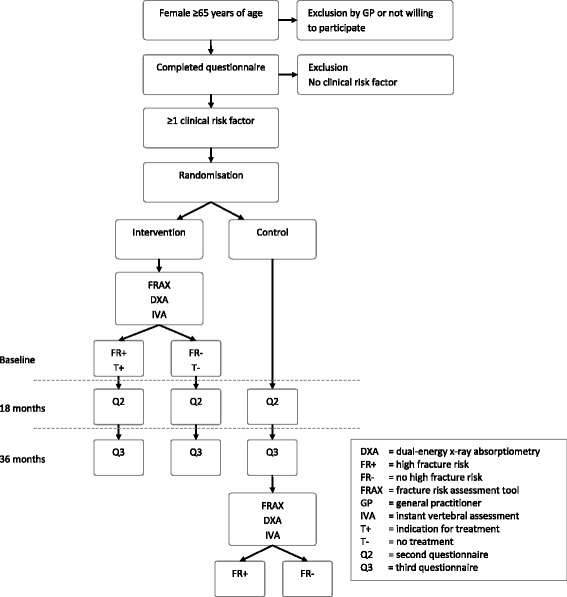
Flow chart of the Salt Osteoporosis Study

The SALT Osteoporosis Study is a randomised pragmatic trial, which is currently performed in the Netherlands and that uses a stepwise approach (Fig. 1). GP’s in the regions of Waterland, Amstelveen, Amsterdam, Haarlem and the Zaan district, in the province of Noord-Holland, are selected to collaborate in the trial. From the databases of the participating GP’s, all women aged ≥65 years are selected.

**Fig. 2:**
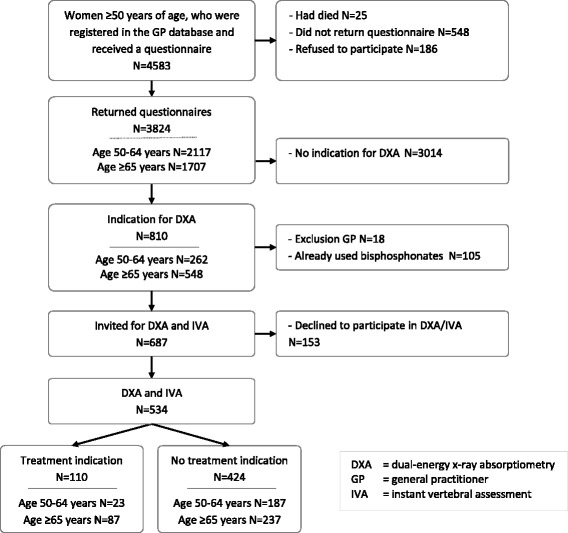
Flow chart of the Pilot SALT Osteoporosis Study in 9 general practices in the Netherlands. The indication of bone densitometry (DXA) and instant vertebral assessment (IVA) was based on the previous Dutch guideline for GP’s for primary osteoporosis (see Table 1)

The decision to include only women aged ≥65 years was based on our pilot study in 9 GP practices (see Fig. 2). In our pilot, women aged ≥50 years (*n* = 3824) were evaluated according to the Dutch conservative guideline. We observed that in women between the age of 50 and 65 years only 1% had an indication for prescription of bone sparing drugs in a primary care setting.

For the SALT Osteoporosis Study, women having terminal illness and women deemed unable to comply with the study protocol are excluded by the GP’s. All other women are invited to participate by their GP and all receive a general baseline questionnaire from the research team (see Table 2 for content). Based on this questionnaire, women using bone sparing drugs or corticosteroids are also excluded (see Table 3 for full exclusion criteria).

**Table 2:** Content of the questionnaires of the SALT Osteoporosis Study

Theme	Questionnaire
Fractures	Q1, Q2, Q3
Examination for osteoporosis	Q1, Q2, Q3
Hip fractures of close family	Q1, Q2, Q3
Falls in last 12 months	Q1, Q2, Q3
Fear of falling	Q1, Q2, Q3
Mobility and use of walking aid	Q1, Q2, Q3
Height and weight	Q1, Q2, Q3
Living situation	Q1, Q2, Q3
Education level	Q1
Ethnicity	Q1
Age of start menopause	Q1
Smoking and alcohol use	Q1, Q2, Q3
Use of dairy products	Q1, Q2, Q3
Use of vitamin D/ calcium/ multi-vitamin supplements	Q1, Q2, Q3
Medication use	Q1, Q2, Q3
Use of osteoporosis medication	Q1, Q2, Q3
Prednison use	Q1
Chronic diseases	Q1, Q2, Q3
Quality of life (EuroQoL-5D)	Q2, Q3
Consultation physician	Q2, Q3
Hospital admission	Q2, Q3
Dizziness	Q2
Incontinence	Q2
Memory complaints	Q2
Depression and anxiety (PHQ-4)	Q2
Attitude toward osteoporosis and perceived risks	Q2
Adherence and persistence to use osteoporosis medication (ADEOS-12)	Q2^a^
Side effects of osteoporosis medication	Q2^a^
Independence	Q3
Diabetes treatment	Q3^b^
Diabetes complications and hypoglycaemic attacks	Q3^b^
Sunlight exposure	QD^c^
Vitamin D via diet and supplements	QD^c^

*Q1* baseline questionnaire, *Q2* questionnaire after 18 months, *Q3* questionnaire after 36 months, *QD* = vitamin D questionnaire. ^a^These items are questioned only to patients with an indication for treatment. ^b^These items are questioned only to patients with diabetes mellitus. ^c^This questionnaire has been send to a random sample of the first participants

**Table 3:** Exclusion criteria of the SALT Osteoporosis Study

Exclusion criteria
Age ≥ 91 years^a^
Actual use of bone sparing drugs^b^
Use of bone sparing drugs during the preceding 5 years
Terminal illness
Not being able to participate in the study or no consent
Weight > 135 kg^c^
Corticosteroid use of ≥7.5 mg prednison equivalent per day

^a^Calculation of FRAX (fracture risk assessment tool) is not possible for this age category

^b^Bisphosphonates, strontiumranelate, estrogens, selective estrogen receptor modulators, parathyroid hormone analogues or denosumab. If new bone sparing drugs will become available during the trial, they were added to the exclusion list

^c^Maximum weight for dual-energy x-ray absorptiometry measurement

The next step beholds that we determine clinical risk factors for fractures that are reported in the questionnaire in all eligible women. The clinical risk factors are based on the fracture risk assessment tool (FRAX) guideline (Table 4) [17–20]. One of the clinical risk factors used in the FRAX is not applied, i.e. long term untreated hyperthyroidism, as it is impossible to find these patients when using a questionnaire, and this condition being very rare in this patient group. The items alcohol use and smoking are left out as well in the selection of participants (they are not excluded from the final FRAX calculation), because their association with fracture risk is relatively weak [21, 22]. After the risk factors are determined, those women who have at least one clinical risk factor for fractures are assigned to the intervention or control group, using individual randomisation.

**Table 4:** Clinical risk factors for fractures used in the SALT Osteoporosis Study

Risk factors^a^
Previous fracture >50 years of age
Parent with hip fracture
Low body weight (BMI <19 kg/m^2^)
Rheumatoid arthritis
Early menopause (<45 years of age)
Malabsorption syndrome
Chronic liver disease
Type I diabetes mellitus
Immobility (severe walking difficulties and/or use of walking aid)

*BMI* body mass index, *COPD* chronic obstructive pulmonary disease, *FRAX* fracture risk assessment tool

^a^Risk factors were derived from the FRAX. We excluded the following risk factors from the FRAX: long term untreated hyperthyroidism, alcohol use and smoking. Women with ≥1 clinical risk factors were randomly assigned to the intervention or control group

In the third step, the women in the intervention group are offered a screening program (as described below), to identify the women with a high fracture risk. Only these women with a high fracture risk are offered a structured treatment program (as described below). Women in the control group receive usual care. Both the control and intervention group are followed for a duration of at least 3 years, by sending follow-up questionnaires at 18 and 36 months (see Table 2 for content, and Table 5 for procedures).

**Table 5:** Procedures of data collection and entry in the SALT Osteoporosis Study

Data collection
Sending of questionnaire by mail
If no reply: repeated sending of questionnaire
If no reply: sending of abbreviated questionnaire
If no reply: follow-up calls by phone
Data entry
Entry of the data in the database in duplo by different administrators
If mismatch: checking of data by third person
If missing data on important questions: follow-up calls by phone If reported rheumatoid arthritis or prednisone use (first questionnaire): verification at general practitioner

The trial is carried out at the VU University Medical Center, Amsterdam, in close collaboration with the Stichting ArtsenLaboratorium and Trombosedienst (SALT), a primary care diagnostic center. All participants provide written informed consent. The trial is approved by the Dutch Health Council (2009/05WBO), and an execution license is provided by the Dutch Ministry of Health, Wellbeing and Sports (PG/)GZ-2.978.265). The trial is registered in the Dutch Trial Register (www.trialregister.nl, NTR2430).

### Screening program

The screening program to identify women with a high fracture risk consists of the FRAX combined with bone mineral density measures (BMD) assessed with DXA, and instant vertebral assessment (IVA), fall risk assessment, and clinical chemistry screening. Details on the measurements procedures are described below.

We have chosen this combined approach to increase the sensitivity to find high risk individuals. The FRAX is an often used tool to identify individuals at high fracture risk [17–20]. The assessment of clinical risk factors in combination with BMD has been shown to be more accurate than BMD alone [23]. IVA has been added as a screening instrument, because it has been shown that elderly with a vertebral fracture have a 4-fold increased fracture risk [24, 25]. In addition, fall risk assessment is not part of the FRAX, but fall-related risk factors are significantly and independently associated with fractures [26]. Almost all wrist fractures and over 90% of the hip fractures are the direct result of a fall [27]. Clinical chemistry is done for the purpose of additional diagnostics to identify secondary osteoporosis.

#### FRAX

The FRAX algorithm calculates a 10-year absolute fracture risk based on the BMD measurement of the hip and the clinical risk factors indicated in the questionnaire. Because a Dutch FRAX tool was not available at the start of the study, the UK FRAX tool is used and adapted thresholds for a high fracture risk are developed based on data from the Longitudinal Aging Study Amsterdam (LASA) [28]. The LASA is a population-based study in a representative sample of the Dutch elderly population. The LASA cohort data was used to calculate the absolute 10-year fracture risk for different age groups using the FRAX, as depicted in Table 6. The absolute threshold for a high fracture risk was defined among women with ≥1 clinical risk factors for fractures.

**Table 6:** Thresholds for treatment calculated using FRAX^a^ and based on LASA cohort data, stratified for age

Age	Total group		No clinical risk factors for fractures		≥1 clinical risk factors for fractures		Treatment threshold
(yrs)	mean FRAX-score (SD)	N	mean FRAX-score (SD)	N	Mean FRAX-score (SD)	N	
65-69	12.1 (5.2)	166	8.9 (1.3)	83	15.3 (5.6)	83	>15%
70-74	14.7 (5.8)	187	10.9 (1.6)	90	18.3 (6.0)	97	>18%
75-79	18.6 (7.6)	154	13.2 (2.0)	76	24.0 (7.2)	78	>24%
80-84	22.1 (8.1)	157	17.3 (2.8)	86	28.0 (8.6)	71	>28%
85-91	26.2 (8.1)	101	19.4 (3.1)	44	31.5 (6.8)	57	>32%

*FRAX* fracture risk assessment tool, *LASA* Longitudinal Aging Study Amsterdam, *SD* standard deviation

^a^UK version of the FRAX tool

#### DXA

DXA measurements are performed at six different locations (Medisch centrum de Vaart Zaandam, Waterlandziekenhuis Purmerend, Kennemer Gasthuis Haarlem, Onze Lieve Vrouwe Gasthuis (OLVG) Amsterdam, Ziekenhuis Amstelland Amstelveen, and Rode Kruis Ziekenhuis Beverwijk). All locations use a Hologic Discovery device (Hologic Inc., USA). BMD of the non-dominant hip and lumbar spine (L1 to L4) are measured under standardized procedures at all locations. In addition, trabecular bone scores are collected [29]. The dominant hip is used in case measurement of the non-dominant hip is not possible, and at least two lumbar vertebrae are used in case examination of L1 to L4 is not possible. The images are evaluated by an analyst and this evaluation is reviewed by a radiologist.

#### IVA

The Hologic Discovery (Hologic Inc., USA) provides a lateral image of the spine under standardized procedures. The IVA is performed according to the semi-quantitative method of Genant [30] by two independent examiners and reviewed by a third examiner in case of discordance between the two examiners. The definition of a vertebral fracture is defined as a height reduction of ≥20% in the lumbar spine and a height reduction of ≥25% in the thoracic spine.

#### Fall risk assessment

Fall risk is assessed with the answers in the initial questionnaire (Table 2). Based on a previously validated risk profile [31], patients who are classified as having increased fall risk have either stated to have fallen twice or more in the previous year, or to have fallen once combined with a reduced mobility or fear of falling (score ≥ 8 (range 1-10, with higher scores indicating more fear of falling)).

#### Clinical chemistry

Blood samples are analyzed routinely at two different locations. At SALT, serum 25-hydroxyvitamin D (25(OH)D) is determined using the DiaSorin chemiluminescent immunoassay (DiaSorin, Italy), until March 2014, the DIAsource enzyme immunoassay until April 2015 (DIAsource ImmunoAssays, Belgium), and the Roche COBAS 6000’s electrochemiluminescence immunoassay thereafter (Roche Diagnostics USA). Serum calcium, creatinine, albumin, thyroxine, and thyroid stimulating hormone (TSH) are determined using the Roche COBAS 6000 from the start (Roche Diagnostics, USA). Erythrocyte sedimentation rate (ESR) is measured in blood on a Ves-Matic Cube (Menarini Diagnostics, Italy).

In the OLVG hospital, for serum 25(OH)D determination, the DiaSorin was used until September 2014 (DiaSorin, Italy), and the Roche COBAS e601/e602 module thereafter (Roche Diagnostics, USA). The Roche COBAS C702 is used to determine serum calcium, creatinine, and albumin (Roche Diagnostics, USA). Thyroxine and TSH are determined on the Roche COBAS e601/602 analyzer (Roche Diagnostics, USA). Blood ESR is measured using the Sysmex Starrsed Interrliner (Sysmex, Japan), that measures ESR according to the Westergren method.

Creatinine clearance is estimated for all participants by the Cockcroft-Gault formula.

### Structured treatment program

Evaluations of the screening program and subsequent formulation of individual treatment advices are the responsibility of an expert team. Precise treatment indications for bone sparing medication are described in Table 7, and the structured treatment program is described in Table 8. The GP’s receive personal instruction about the structured treatment protocol and receive the individual treatment advice for each patient. However, they have professional freedom to decide whether they are going to treat and which medication they are going to use. All GP’s are instructed to report to the research team about each high fracture risk patient in the intervention group if treatment is started and if yes, which treatment is used.

**Table 7:** Treatment indications for bone sparing medication in the SALT Osteoporosis Study

Treatment indications
Lumbar fracture on IVA with a vertebral height reduction ≥20%^a^
Thoracic fracture on IVA with a vertebral height reduction ≥25%^a^
Fracture risk according to FRAX ≥age specific threshold (see Table 6) and DXA (hip and/or spine) T-score ≤ −2
Treatment indication according to the actual Dutch guideline for GP’s for primary osteoporosis (see Table 1)

*DXA* dual-energy x-ray absorptiometry, *FRAX* fracture risk assessment tool, *GP* general practitioners, *IVA* instant vertebral assessment

^a^According to the semi-quantitative technique of Genant [30]

**Table 8:** Structured treatment program of the SALT Osteoporosis Study

Treatment protocol
Calcium supplementation - ≥4 dairy consumptions/day: none - 2-3 dairy consumptions/day: 500 mg calcium/day - 0-1 dairy consumptions/day: 1000 mg calcium/day
Vitamin D supplementation - 20 μg (800 IU)/day if serum 25-hydroxyvitamin D < 50 nm/L
Bone sparing drugs for a duration of 5 years - First choice treatment: alendronate 70 mg/week or risedronate 35 mg/week - Second choice treatment: zoledronic acid 5 mg/year intravenous or denosumab 60 mg subcutaneous/6 months^a^ - Third choice treatment: ibandronic acid 150 mg/month or strontium ranelate 2 mg/day at night (if subject is ≥74 years of age)
Fall prevention - Notification to GP of increased fall risk if ≥2 falls in the last year mentioned in the questionnaire, or 1 fall in combination with immobility, or fear of falling
Additional evaluation by GP or referral to secondary care - ESR >50 mm/h - Calcium (albumin corrected) ≥2.60 mmol/L - TSH <0.3 mU/L and free T4 > 21 pmol/L - TSH >3.7 mU/L and free T4 < 12 pmol/L - Creatinine clearance according to Cockcroft-Gault formula <30 mL/min
Parathyroid hormone analogues - New vertebral fractures after one year treatment with bone sparing drugs with multiple prevalent vertebral fractures

*GP* general practitioner, *ESR* erythrocyte sedimentation rate, *TSH* thyroid stimulating hormone, *T4* thyroxine

^a^Denosumab has been added as an treatment option since the inclusion of denosumab in the updated version of Dutch guidelines for GP’s in October 2012

As specified by the Dutch guidelines, GP’s are instructed to organise a consultation with patients after 4 weeks, 3 months, 6 months, and every year after the start of bone sparing treatment, in order to optimize adherence and to evaluate side effects. For this reason, GP’s are offered an option to use an application to remind them that a contact is due. GP’s report each consult that occurred, and the application notifies the GP’s if these contacts are due or have been missed.

The GP’s are notified if patients in the intervention group have an increased fall risk. The decision whether and which measures to take for fall prevention is not protocolled.

### Control group

Women in the control group receive usual care. At baseline, only clinical risk factors for fractures are examined in the control group, because it has been considered to be unethical to offer women in the control group a DXA without informing them about the results. A waiting list construction is therefore applied to the control group: at the end of the trial, the women in the control group will undergo the same screening as the women in the intervention group.

For additional ethical reasons, all patients in the control group that have an indication for DXA and IVA according to the Dutch guideline for GP’s at the time of their inclusion, are notified accordingly and advised to contact their GP as part of usual care.

### Randomisation

Women with at least one clinical risk factor for fractures, as determined by the baseline questionnaire, are assigned to the intervention or control group using individual randomisation, in a 1:1 ratio. The randomisation sequence is computer-generated using the MT_rand function of Hypertext Preprocessor (PHP). Via our web-based database, group allocation is automatically assigned to every new patient that is included.

### Blinding

Complete blinding of the patients and study team is not feasible in this open study design. However, we took measures to blind the GP as much as possible: the GP only receives information about patients with a treatment indication in the intervention group. For the other patients, the GP does not know whether or not they have approved to participate, are low risk participants in the intervention group, or are assigned to the control group. In addition, data entry and verification of fractures are performed by study team members that are blinded to group allocation. Statistical analyses will not be performed blindly.

### Outcomes

The primary outcome is the time to the first new fracture and the total number of fractures during follow-up. Participants are asked to report fractures at 18 and 36 months, using questionnaires. All fractures will be verified with the GP or the hospital. Fracture information of patients who die during the study, move out of the region, or are otherwise lost to follow-up will be extracted from the GP medical records as well. Additionally, we will study the GP medical records of a subsample of 10% of the patients, who do not report fractures, to estimate the amount of fractures we might have missed using the questionnaires.

Secondary outcomes are osteoporotic fractures (defined as all fractures except fractures of the head, hand/finger, and foot/toe), as well as hip fractures, self-reported falls, and mortality.

### Qualitative evaluation

Data for a qualitative process evaluation are collected by visiting a subsample of GP practices, patients, and pharmacies. Via interviews, medical records and pharmacy data we will gain insight in the process and anticipate on potential problems.

### Statistical methods

Cox proportional Hazards Models will be used to assess the effect on time to first new fracture between the groups. The proportional hazard assumption will be evaluated. The primary analyses will contain two different comparisons. First, to examine the effect of the screening program plus the structured treatment program, the women in the intervention group will be compared with the women in the control group. Following the intention to treat principle, this comparison includes all randomised women, both the ones with a high fracture risk and the ones with a low fracture risk. Second, we will examine the effect of the structured treatment program. In this analyses, women with a high fracture risk as identified by the screening program and adjoined treatment indication in the intervention group will be compared to women with a high fracture risk and adjoined treatment indication in the control group.

For the latter comparison, data on the fracture risk at baseline will be missing for most of the women in the control group, because DXA and IVA measurements are only performed at the end of 3 years of follow-up in the majority of the controls. In controls who underwent DXA and IVA at baseline, the baseline data will be used. Otherwise, missing data on fracture risk will be substituted. If BMD at follow-up will be sufficient, it is assumed that it was sufficient at baseline too, and if no vertebral fractures are observed at follow-up, than it is likely that no vertebral fractures were present at baseline either. The remainder of missings will be substituted using multiple imputation. For multiple imputation, the available baseline data of the intervention and control group will be used to estimate fracture risk for the women with remaining missing data.

In a secondary analysis we will examine the per protocol effect among adherent women only. All analyses will be adjusted for variables that differ between the intervention and control group at baseline. Interaction effects with age and previous fracture will be examined. An interim analysis has been performed to see if the study needed to be extended or stopped for futility. This analysis did not have consequences for the study continuation.

### Sample size calculation

In our pilot study, 3% of all participants fulfilled the selection criteria for treatment with bone sparing drugs according to the actual GP guideline (Fig. 2). Based on the questionnaires we calculated the absolute 10-years fracture risk of the three main types of osteoporotic fractures using the FRAX. Based on this FRAX calculation and using the threshold levels of Table 6, we estimated that 28.2% of the women would fulfil criteria for treatment and that the average absolute 10-years fracture risk of these women was 27.5%, or 8.3% in a 3-year follow-up.

We assume that the incidence of new fractures will be reduced with 35% in the high risk treatment group compared to the control group [5, 6], and aim for an α of 0.05 and a β of 0.2. Taking into account that 3% of the high risk patients in the control group fulfil criteria for treatment with bone sparing drugs based on the current Dutch guidelines, and allowing for a 30% lost to follow-up rate [extrapolated from 26], we calculated that we would need to include *n* = 1700 in the intervention group and in the control group in order to have *n* = 1190 patients in both treatment groups at the end of the trial.

## Results of inclusion

The inclusion started in 2010. After one year of recruitment, the inclusion was disappointing: only 50% of the approached patients approved to participate instead of the anticipated 80%, and less participants were identified as having a high fracture risk. As a result, more general practices are needed than the 100 practices that were planned, as well as more DXA examinations.

## Discussion

Because of the pragmatic study design the results of the SALT Osteoporosis Study will be very important for underpinnings of the prevention strategy of the osteoporosis guidelines. Currently, another large pragmatic trial to the effectiveness of screening older women for the prevention of fractures (the SCOOP study) is being performed in the United Kingdom [29]. The screening strategy of SCOOP is fairly similar to our strategy. In SCOOP, the indication for DXA is determined by the 10-year probability of a hip fracture as calculated by FRAX. After the DXA measurement in women at high risk, the 10-year probability is recalculated following inclusion of femoral neck BMD, to select the women that should be treated. In contrast to our study, IVA is not part of the screening program. The results of the SCOOP trial, are being expected in the coming year.

In addition to its pragmatic design and the use of DXA and IVA, the strength of the current study is that it is being performed in a primary care setting. We will therefore be able to check the follow-up status on fractures and mortality of patient who will be lost during follow up in the medical files, and as a consequence, low rates of missing data on the outcome are expected.

A limitation is the waiting list control group, in which the controls will undergo DXA after the end of the trial. This was done because of ethical considerations. However, inevitably, participants will be lost to follow-up, and therefore missing data on DXA will be increased in the control group, as compared to the intervention group. Multiple imputation will be used to solve this problem.

The sample size calculation was made to examine the effect of the structured treatment program, not primarily to examine the effect of the screening plus treatment program. Therefore, the power to show an effect in the intention to treat analysis (effect of screening and treatment program) is probably not large enough, because of a limited contrast between the treatment groups in this analysis: both the intervention and the control group contain women that do not have an indication for treatment. A larger sample size was practically and financially not feasible. In addition, the sample size calculation was based on the incidence of the three main types of osteoporotic fractures, whereas the main outcome is all types of fractures. The reason for this is that the FRAX algorithm was used to estimate fracture incidence, and this tool is not available for all types of fractures. The choice for all fractures as primary outcome results in 20% more fractures [32], and therefore increases the statistical power. Osteoporotic fractures will be analyzed subsequently.

If our screening program is effective, it might reduce the cost of fractures, but will also bring additional costs for screening and treatment. A cost-effectiveness evaluation is therefore of major interest. However, only a restricted cost-effectiveness analysis is possible for this study. Patients will be asked about medical visits and hospital admission twice during the follow-up of three years. We will be able to estimate and compare the costs of a fracture in both treatment arms.

In the Netherlands, usual osteoporosis care is problem-driven in reaction to, for example, a fracture or a question of the patient. Standardized screening in which x-ray examination is part of the process, is not allowed without special permit by the government. We are performing the study as a population study, with approval of the Dutch Health Council and Dutch Ministry of Health, Wellbeing and Sports, in which all women of 65 years or older are invited to participate and we will perform a process analysis of this approach by interviewing patients, doctors and general practices. This might lead to additional insights on how the prevention of osteoporosis in primary care can be improved. The primary result of the SALT Osteoporosis Study, that is the effectiveness of the screening and treatment program, will have a large impact on the strategy for fracture prevention.

## Abbreviations

25(OH)D: 25-hydroxyvitamin D
BMD: Bone mineral density
DXA: Dual x-ray absorptiometry
ESR: Erythrocyte sedimentation rate
FRAX: Fracture risk assessment tool
GP: General practitioner
IVA: Instant vertebral assessment
LASA: Longitudinal Aging Study Amsterdam
SALT: Stichting ArtsenLaboratorium en Trombosedienst
TSH: Thyroid stimulating hormone

## References

[1] Genant HK, Cooper C, Poor G (1999). Interim report and recommendations of the World Health Organization task-force for osteoporosis. Osteoporos Int.

[2] Seeley DG, Browner WS, Nevitt MC, Genant HK, Scott JC, Cummings SR (1991). Which fractures are associated with low appendicular bone mass in elderly women? The study of osteoporotic fractures research group. Ann Intern Med.

[3] Lotters FJ, van den Bergh JP, de Vries F, Rutten-van Molken MP (2016). Current and future incidence and costs of osteoporosis-related fractures in The Netherlands: combining claims data with BMD measurements. Calcif Tissue Int.

[4] Lips P, van Schoor NM (2005). Quality of life in patients with osteoporosis. Osteoporos Int.

[5] Wells G, Cranney A, Peterson J, Boucher M, Shea B, Robinson V, Coyle D, Tugwell P. Risedronate for the primary and secondary prevention of osteoporotic fractures in postmenopausal women. Cochrane Database Syst Rev. 2008;1:CD004523. 10.1002/14651858.CD004523.pub3 18254053

[6] Wells GA, Cranney A, Peterson J, Boucher M, Shea B, Robinson V, Coyle D, Tugwell P. Alendronate for the primary and secondary prevention of osteoporotic fractures in postmenopausal women. Cochrane Database Syst Rev. 2008;1:CD001155. 10.1002/14651858.CD001155.pub2 18253985

[7] Wells GA, Cranney A, Peterson J, Boucher M, Shea B, Robinson V, Coyle D, Tugwell P. Etidronate for the primary and secondary prevention of osteoporotic fractures in postmenopausal women. Cochrane Database Syst Rev. 2008;1:CD003376. 10.1002/14651858.CD003376.pub3 PMC6999803 18254018

[8] Serrano AJ, Begona L, Anitua E, Cobos R, Orive G (2013). Systematic review and meta-analysis of the efficacy and safety of alendronate and zoledronate for the treatment of postmenopausal osteoporosis. Gynecol Endocrinol.

[9] Briot K, Cortet B, Thomas T (2012). 2012 update of French guidelines for the pharmacological treatment of postmenopausal osteoporosis. Joint Bone Spine.

[10] Compston J, Bowring C, Cooper A (2013). Diagnosis and management of osteoporosis in postmenopausal women and older men in the UK: National Osteoporosis Guideline Group (NOGG) update 2013. Maturitas.

[11] Elders PJM, Dinant GJ, van Geel T, Maartens LWF, Merlijn T, Geijer RMM, Geraets JJXR (2012). NHG-Standaard Fractuurpreventie (tweede herziening). Huisarts Wet.

[12] Kanis JA, McCloskey EV, Johansson H, Cooper C, Rizzoli R, Reginster JY (2013). European guidance for the diagnosis and management of osteoporosis in postmenopausal women. Osteoporos Int.

[13] Mendoza N, Sanchez-Borrego R, Villero J (2013). 2013 up-date of the consensus statement of the Spanish menopause society on postmenopausal osteoporosis. Maturitas.

[14] Watts NB, Bilezikian JP, Camacho PM (2010). American Association of Clinical Endocrinologists Medical Guidelines for clinical practice for the diagnosis and treatment of postmenopausal osteoporosis. Endocr Pract.

[15] Yeap SS, Hew FL, Lee JK, Goh EM, Chee W, Mumtaz M, Damodaran P, Lim HH, Chan SP (2013). The Malaysian clinical guidance on the management of postmenopausal osteoporosis, 2012: a summary. Int J Rheum Dis.

[16] Patsopoulos NA (2011). A pragmatic view on pragmatic trials. Dialogues Clin Neurosci.

[17] Centre for Metabolic Diseases, University of Sheffield, UK. https://www.shef.ac.uk/FRAX/index.aspx. Accessed 3 Jan 2017.

[18] Kanis JA, Johnell O, Oden A, Johansson H, McCloskey E (2008). FRAX and the assessment of fracture probability in men and women from the UK. Osteoporos Int.

[19] Kanis JA, McCloskey EV, Johansson H, Oden A, Strom O, Borgstrom F (2010). Development and use of FRAX in osteoporosis. Osteoporos Int.

[20] Kanis JA, McCloskey EV, Johansson H, Strom O, Borgstrom F, Oden A (2008). Case finding for the management of osteoporosis with FRAX--assessment and intervention thresholds for the UK. Osteoporos Int.

[21] Kanis JA, Johansson H, Johnell O, Oden A, De Laet C, Eisman JA, Pols H, Tenenhouse A (2005). Alcohol intake as a risk factor for fracture. Osteoporos Int.

[22] Kanis JA, Johnell O, Oden A (2005). Smoking and fracture risk: a meta-analysis. Osteoporos Int.

[23] Kanis JA, Oden A, Johnell O (2007). The use of clinical risk factors enhances the performance of BMD in the prediction of hip and osteoporotic fractures in men and women. Osteoporos Int.

[24] Pongchaiyakul C, Nguyen ND, Jones G, Center JR, Eisman JA, Nguyen TV (2005). Asymptomatic vertebral deformity as a major risk factor for subsequent fractures and mortality: a long-term prospective study. J Bone Miner Res.

[25] Black DM, Arden NK, Palermo L, Pearson J, Cummings SR (1999). Prevalent vertebral deformities predict hip fractures and new vertebral deformities but not wrist fractures. Study of Osteoporotic Fractures Research Group. J Bone Miner Res.

[26] CBO KvdG (2011) Richtlijn Osteoporose en fractuurpreventie, derde herziening.

[27] Parkkari J, Kannus P, Palvanen M, Natri A, Vainio J, Aho H, Vuori I, Jarvinen M (1999). Majority of hip fractures occur as a result of a fall and impact on the greater trochanter of the femur: a prospective controlled hip fracture study with 206 consecutive patients. Calcif Tissue Int.

[28] Hoogendijk EO, Deeg DJ, Poppelaars J (2016). The longitudinal aging study Amsterdam: cohort update 2016 and major findings. Eur J Epidemiol.

[29] Martineau P, Leslie WD. Trabecular bone score (TBS): Method and applications. Bone. 2017;104:66–72. 10.1016/j.bone.2017.01.035 28159710

[30] Genant HK, Wu CY, van Kuijk C, Nevitt MC (1993). Vertebral fracture assessment using a semiquantitative technique. J Bone Miner Res.

[31] Peeters GM, Elders PJM, Lips P, Deeg DJ (2011). Snelle inschatting van de kans op herhaald vallen bij ouderen. Huisarts Wet.

[32] van Wijngaarden JP, Swart KM, Enneman AW (2014). Effect of daily vitamin B-12 and folic acid supplementation on fracture incidence in elderly individuals with an elevated plasma homocysteine concentration: B-PROOF, a randomized controlled trial. Am J Clin Nutr.

